# Sex Differences in Genetic Effects Using Two Gene Aggregation Techniques: Depression and Cognition

**DOI:** 10.1093/geroni/igaa057.2978

**Published:** 2020-12-16

**Authors:** Erin Ware, Lauren Schmitz, Arianna Gard

**Affiliations:** 1 University of Michigan, Ann Arbor, Michigan, United States; 2 University of Wisconsin, Madison, Wisconsin, United States

## Abstract

The state of science has increasingly valued interdisciplinary training and research. As an early-stage investigator, interdisciplinary research, such as that supported by the Research Centers Collaborative Network through the National Institute on Aging, provides valuable opportunities to connect researchers across disciplinary boundaries. This program encourages researchers use their diverse training experiences to tackle difficult questions in aging research and forge scholarly relationship that advance the state of science through creative problem-solving and a spectrum of interdisciplinary perspectives. This presentation describes a recently funded project to examine sex differences in aging outcomes using genetics, depression, and impaired cognition with polygenic and gene-region aggregation techniques. We will highlight the benefits and opportunities of the Research Centers Collaborative Network pilot grant to this highly interdisciplinary work, co-lead by early-stage investigators.

